# Trichobezoar Causing Airway Compromise during Esophagogastroduodenoscopy

**DOI:** 10.1155/2015/806857

**Published:** 2015-09-17

**Authors:** Erica Y. Kao, Nicholas J. Scalzitti, Gregory R. Dion, Sarah N. Bowe

**Affiliations:** ^1^San Antonio Military Medical Center Uniformed Services Health Education Consortium, 3551 Roger Brooke Drive, JBSA-Fort Sam Houston, TX 78234, USA; ^2^Uniformed Services University of the Health Sciences, 4301 Jones Bridge Road, Bethesda, MD 20814, USA

## Abstract

*Objectives*. (1) Report the case of a 5-year-old female with trichotillomania and trichophagia that suffered airway compromise during esophagogastroduodenoscopy for removal of a trichobezoar. (2) Provide management recommendations for an unusual foreign body causing extubation and partial airway obstruction. *Methods*. Case report of a rare situation of airway compromise caused by a trichobezoar. *Results*. A 5-year-old patient underwent endoscopic retrieval of a gastric trichobezoar (hairball) by the gastroenterology service under general endotracheal anesthesia in a sedation unit. During removal, the hairball, due to its large size, dislodged the endotracheal tube, effectively extubating the patient. The bezoar became lodged at the cricopharyngeus muscle. Attempts to remove the bezoar or reintubation were unsuccessful. The child was able to be mask ventilated while the otolaryngology service was called. Direct laryngoscopy revealed a hairball partially obstructing the view of the glottis from its position in the postcricoid area. The hairball, still entrapped in the snare from the esophagoscope, was grasped with Magill forceps and slowly extracted. The patient was then reintubated and the airway and esophagus were reevaluated. *Conclusions*. Trichobezoar is an uncommon cause of airway foreign body. Careful attention to airway management during these and similar foreign body extractions can prevent inadvertent extubations.

## 1. Introduction

Children and adolescents with a history of trichotillomania and trichophagia can present with trichobezoars, which are accumulations of hair in the stomach secondary to impulsive hair-pulling and consumption [1]. The term trichobezoar originates from “trich” (Greek for hair) and “bezoar” (Arabic and Persian for poison antidote) [2]. On rare occasions, the trichobezoar may have a tail of hair extending beyond the stomach into the duodenum and jejunum, known as Rapunzel syndrome [3]. Common presenting symptoms include chronic abdominal pain, gastric outlet obstruction, nausea, vomiting, weight loss, malnutrition, hematemesis, diarrhea, and constipation [2]. Trichobezoars are treated with either endoscopic removal with or without mechanical fragmentation or open surgical treatment consisting of laparotomy and gastrostomy under general anesthesia [4]. Previous studies have identified that the location and size of the bezoar have a direct impact on treatment modality, as well as overall success of the attempt [4]. The endoscopic approach is ideal for favorably sized trichobezoars (i.e., <6 cm) located within or proximal to the stomach [4, 5]. Park et al. compared clinical outcomes of patients with trichobezoars who underwent endoscopies and found that multiple endoscopic procedures were needed for bezoars greater than 6 cm [4]. Surgical removal remains necessary for large trichobezoars and Rapunzel syndrome with extension into the small bowel [4, 6]. Case reports exist documenting unsuccessful attempts at endoscopic removal necessitating surgical extraction of the bezoar [5]. We report the case of a patient with a trichobezoar removed by endoscopy with extubation occurring as a result of the procedure. By recognizing this potential outcome, specific modifications in technique can reduce the likelihood for its occurrence in future endoscopic procedures.

## 2. Case Report

**Figure 1:**
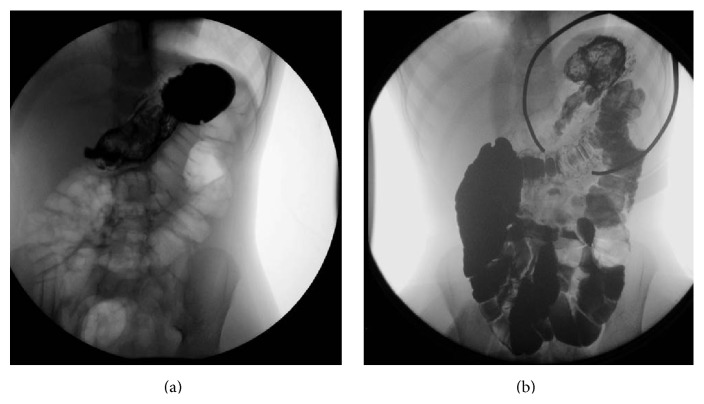
2012 upper GI study showing gastric bezoar (a); 2014 upper GI study showing gastric bezoar (b).

A 5-year-old female with a history of trichotillomania and trichophagia presented to the pediatric gastroenterology clinic with the chief complaints of abdominal pain, alopecia, and passing hair in the stool. Her mother noticed her habit of trichotillomania and trichophagia 3 years before with variable severity and frequency. The patient was noted to have several areas on her scalp of thinning hair. Besides consuming her own hair, the girl was also noted to eat Barbie doll hair, stuffed animal hair, and hair off the ground. The patient had previously presented at age 3 with abdominal pain, progressively poor oral intake, diarrhea, and emesis secondary to a gastric trichobezoar, confirmed on upper GI barium study, which required endoscopic removal (Figure 1). The bezoar was large enough to necessitate piecemeal extraction before it could be effectively retrieved out of the stomach and esophagus. The patient's recurrence of trichobezoar was confirmed by contrasted upper GI series and a small bowel follow-through revealing a mobile, mottled filling defect in the stomach (Figure 1). There was no evidence of extension into the small bowel.

**Figure 2:**
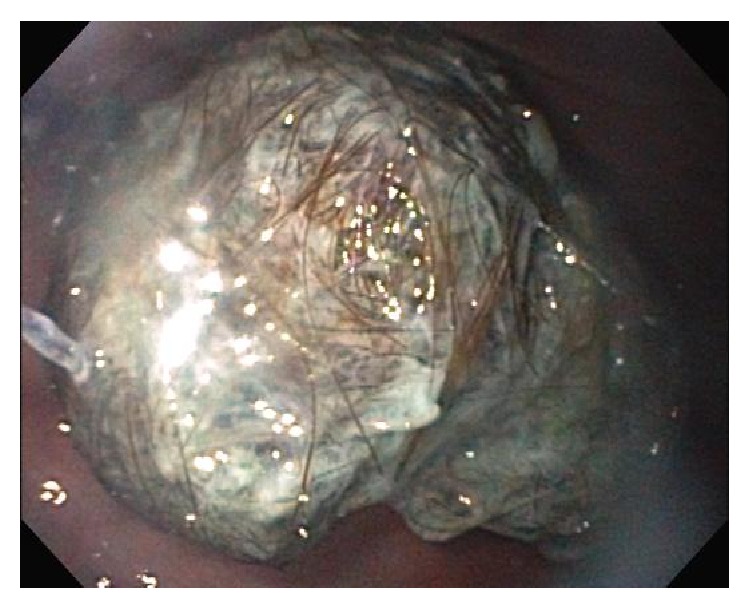
Trichobezoar visualized on endoscopy.

**Figure 3:**
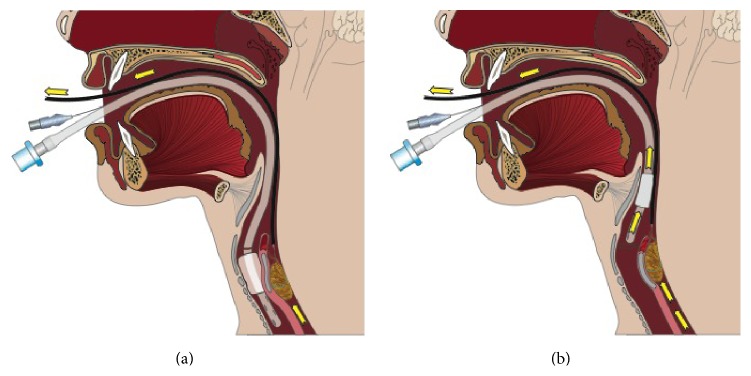
Mass effect of trichobezoar being pulled up (a) and pushing against the posterior tracheal wall causing extubation (b).

The patient was taken to the operating room for endoscopic retrieval of the bezoar (Figure 2). During the procedure, the mass caught at the cricopharyngeus, causing the endotracheal tube to dislodge. More specifically, the bezoar in the esophagus pressed anteriorly on the posterior tracheal wall and created a mass effect (Figure 3). As the bezoar reached the level of the cuffed endotracheal tube, it pushed the balloon on the endotracheal tube superiorly and extubated the patient. At this point, the trichobezoar became lodged at the level of the cricopharyngeus. Initial attempts to remove the bezoar or reintubation were unsuccessful. Fortunately, the child was able to be mask ventilated while the otolaryngology service was called. Direct laryngoscopy and evaluation with rigid telescopes revealed a hairball still affixed to the endoscopic snare. The mass was 4 cm across and had taken on a dumbbell shape with a component deep to the cricopharyngeus and the remainder partially obstructing the view of the glottis from its position in the postcricoid area of the hypopharynx. There was no evidence of hemorrhage or trauma to the hypopharynx or larynx from the attempted foreign body removal and inadvertent extubation. After a careful anatomic assessment, the bezoar and entrapped snare were removed using Magill forceps in a hand-over-hand technique using slow, gentle pressure. No attempts were made at piecemeal removal as the bezoar was completely encased in the snare. The patient was then reintubated with a 4.5 endotracheal tube for a more comprehensive aerodigestive tract evaluation. Examination with a rigid endoscope showed moderate supraglottic edema, while the glottis appeared normal. The patient was able to be extubated without issues, and a 0.5 mg/kg dose of intravenous dexamethasone was given. The child was observed for several hours following the procedure and did not develop any airway distress or stridor. She was discharged home the same day.

## 3. Discussion

Trichobezoars are accumulations of foreign material in the stomach or intestines. They have been a topic of interest for years. As early as the twelfth century BC, bezoars were thought to be remedies against various poisons and diseases. Epidemiologically, trichobezoars are seen most commonly in older girls and adult women with psychiatric illness and trichophagia [7]. One study compared a group of patients between the ages of 18–26 and found a female to male ratio of 4 : 1 in younger adults to 15 : 1 in older patients [8]. Clinically, trichobezoars present with symptoms of vague upper abdominal pain, weight loss, food intolerance, occasional vomiting, and palpable abdominal mass [9]. If the hairball is near the pylorus, pyloric stenosis-like symptoms may result with paroxysmal vomiting and epigastric pain [9]. Serious complications of hairballs can occur such as ulceration, obstruction, bleeding, perforation, and peritonitis [9, 10]. Grosfeld and colleagues noted that characteristic findings on upper gastrointestinal contrast studies for cases of gastric trichobezoars included a mass with shaggy edges and a mottled center [7].

Extraction of the hairball using chemical or mechanical fragmentation is the preferred treatment. Adjuvant physical precautions and psychological evaluation are critical postextraction precautions to prevent further ingestion of hair [9, 10]. Small bezoars can be removed nonsurgically by endoscopy and gradual fragmentation; however, large bezoars require laparoscopy [8]. In cases of Rapunzel syndrome with giant bezoars, removal can require laparotomy and an incision in the affected area of the gastrointestinal tract [8]. Advances in technology and instruments allow endoscopic removal of bezoars more commonly than other more invasive, surgical approaches [4]. Challenges still arise with large bezoars. For example, the bezoar may become lodged in the posterior cricoid area during endoscopic retrieval resulting in extubation or airway obstruction, necessitating prompt intervention. In the present case, the trichobezoar became lodged at the cricopharyngeus muscle and dislodged the endotracheal tube. For this patient, a laryngoscope was used to visualize the bezoar and Magill forceps were used to remove it.

There is little published on the topic of airway compromise during endoscopic removal of trichobezoars, but we should follow principles from other similar foreign body situations when managing the airway. Bao noted rapid and effective extraction with throat-operating forceps (Magill forceps) in cases of children with foreign bodies that were blunt, located in the upper esophagus, and not embedded in the esophageal wall [11]. These principles include performing extraction under endotracheal intubation, optimizing the general condition of the patient before the procedure, having readily available equipment (e.g., endotracheal tubes, suction equipment, and first aid medication), selecting size and type of intubating forceps based on the situation, avoiding dental and pharyngeal hemorrhage when using a laryngoscope, and shortening procedure duration to minimize severity of obstruction and tracheal compression [11]. In addition, throat-operating forceps are advantageous in endoesophageal foreign body removal due to their flexible forceps clamps, smooth distal ends that do not injure mucus membranes, and different sizes that are suitable for patients of different ages [11].

There are additional modalities in the literature that have proven useful in endoscopic removal of larger bezoars. The use of proteolytic enzymes or even cola to partially dissolve a bezoar mass has been reported [4]. Cola is thought to aid in chemical dissolution of bezoars due to the acidity of carbonic and phosphoric acid, enhanced by the carbon dioxide bubbles and the mucolytic effect of sodium bicarbonate contained within the beverage [4]. Especially for cases of hard and large bezoars, or even for cases of Rapunzel syndrome where the trichobezoar has an extension distal to the stomach, gradual dissolution by repeated administration of cola followed by endoscopic lithotripsy could minimize complications due to mass effect.

Reevaluating the current case, endoscopic retrieval affords the opportunity to visualize the region of the esophagus as the trichobezoars are being removed. As the cervical esophagus is approached, the air could be removed from the endotracheal balloon temporarily. This would reduce the diameter of the tube and the likelihood for mass effect to be exerted at the bottom of the balloon, forcibly extubating the patient. Once the trichobezoar bypasses the cricopharyngeus, the balloon can be reinflated for the remaining portions of the case.

## 4. Conclusions

In patients with a history of trichotillomania and trichophagia, problems caused by trichobezoars may occur. Endoscopic removal of blunt upper esophageal foreign bodies such as trichobezoars carries the risk of airway complications such as extubation due to bezoar size. It is important to stay vigilant in airway management during endoscopic removal of foreign bodies. Delivery of proteolytic enzymes or cola prior to the procedure can help reduce the size of the bezoar. Deflating the endotracheal tube cuff can reduce the possibility of mass effect resulting in extubation. Finally, one should consider having tools for proper visualization, such as a laryngoscope, and foreign body removal, such as throat-operating forceps (e.g., Magill forceps), readily available in case of emergency.
